# Influences of genetic variants on stroke recovery: a meta-analysis of the 31,895 cases

**DOI:** 10.1007/s10072-019-04024-w

**Published:** 2019-07-29

**Authors:** Nikhil Math, Thang S. Han, Irina Lubomirova, Robert Hill, Paul Bentley, Pankaj Sharma

**Affiliations:** 1 Department of Neuroscience, Imperial College London, South Kensington, London, SW7 2AZ UK; 2 Institute of Cardiovascular Research Royal Holloway, University of London, Egham, Surrey TW20 0EX UK; 3 Department of Endocrinology, Ashford & St Peter’s NHS Foundation Trust, Chertsey, England; 4 Imperial College Healthcare NHS Trust, London, W2 1NY UK

**Keywords:** Polymorphisms, *POE*, *BDNF*, Stroke outcomes

## Abstract

**Background:**

The influences of genetic variants on functional clinical outcomes following stroke are unclear. In order to reliably quantify these influences, we undertook a comprehensive meta-analysis of outcomes after acute intracerebral haemorrhage (ICH) or ischaemic stroke (AIS) in relation to different genetic variants.

**Methods:**

PubMed, PsycInfo, Embase and Medline electronic databases were searched up to January 2019. Outcomes, defined as favourable or poor, were assessed by validated scales (Barthel index, modified Rankin scale, Glasgow outcome scale and National Institutes of Health stroke scale).

**Results:**

Ninety-two publications comprising 31,895 cases met our inclusion criteria. Poor outcome was observed in patients with ICH who possessed the *APOE4* allele: OR =2.60 (95% CI = 1.25–5.41, *p* = 0.01) and in AIS patients with the *GA* or *AA* variant at the *BDNF-196* locus: OR = 2.60 (95% CI = 1.25–5.41, *p* = 0.01) or a loss of function allele of *CYP2C19*: OR = 2.36 (95% CI = 1.56–3.55, *p* < 0.0001). Poor outcome was not associated with *APOE4*: OR = 1.02 (95% CI = 0.81–1.27, *p* = 0.90) or *IL6-174 G/C*: OR = 2.21 (95% CI = 0.55–8.86, *p* = 0.26) in patients with AIS.

**Conclusions:**

We demonstrate that recovery of AIS was unfavourably associated with variants of BDNF and CYP2C19 genes whilst recovery of ICH was unfavourably associated with *APOE4* gene.

**Electronic supplementary material:**

The online version of this article (10.1007/s10072-019-04024-w) contains supplementary material, which is available to authorized users.

## Introduction

After stroke, the majority of patients achieve fastest recovery by 3 months followed by a deceleration and plateauing thereafter [1, 2]. Whilst many factors may influence functional recovery including the size and location of the lesion, delay in treatment and age, these factors do not entirely explain the variability in outcome of stroke [2, 3].

Animal studies have demonstrated an influential role for genetics in post-stroke recovery [4]. A number of genes have been implicated which may impact anywhere from a molecular level upwards, culminating in anatomical changes seen with the degree of axonal sprouting and the strength of subsequent adaptive connections [5]. *BDNF* (brain-derived neurotrophic factor) is one such gene in which its higher concentrations being observed to correlate with favourable outcome in murine models [6].

A previous meta-analysis of stroke patients [7] has been conducted to quantify the specific role of the gene apolipoprotein E (*APOE*) but found no correlation with outcomes in patients recovering from AIS but appeared to associate with worse outcomes in those recovering from ICH (albeit with one study). Hitherto, there has been no attempt to quantify the roles of other genes. We have therefore conducted a meta-analysis to examine the associations of genetic variants with functional outcomes in patients recovering from AIS or ICH. To the best of our knowledge, this is the largest such study to date.

## Methods

### Literature search

We searched electronic databases including EMBASE, Medline, PsycINFO and PubMed up to and including January 2019 using search terms ‘cerebral infarct’, ‘stroke’, ‘brain vascular accident’, ‘cerebrovascular accident’ and ‘intracerebral haemorrhage’. Abbreviations (e.g. ICH) and MeSH terms were also searched. The terms ‘genetic’, ‘polymorphism’, ‘variant’, ‘variation’, ‘mutation’, ‘genotype’ or ‘phenotype’ were searched to identify genetic variants. Stroke outcomes were searched using the terms ‘NIHSS’, ‘Barthel’, ‘Rankin’, ‘Glasgow’, ‘Fugl-Meyer’, ‘FIM’ or ‘outcome’. These terms were combined using AND/OR Boolean operators. Additionally, references of all included publications were examined. If multiple publications on the same data were discovered, the largest dataset was selected. Non-English publications were also included.

### Selection criteria

Studies describing patients with different genetic variants and their functional outcome at the latest available time point were included. Where multiple time points were measured, the final point was used. Where investigators did not detail rehabilitation strategies, it was assumed patients received rehabilitation according to local guidelines. Only studies in adults (aged ≥ 18 years) were selected. Monogenic stroke disorders were excluded as was transient ischaemic attacks and subarachnoid haemorrhage.

### Statistical analysis

Genetic factors including *GA* or *AA* variant at the *BDNF-196* locus, loss of function allele of *CYP2C19*, *E4* allele and *IL6* were used as determinant factors and Barthel index (BI), the modified Rankin scale (mRS), Glasgow Outcome Score (GOS) and the National Institutes of Health Stroke Scale (NIHSS) were used as stroke functional outcomes.

Only variants of the genes *APOE*, *BDNF-196*, *CYP2C19* and *IL6* were examined whilst the remaining genes and polymorphisms were not included due to insufficient number of studies or suitable data. McCarron et al. [8] did not use a validated scale whilst Broderick et al. [9] classified *APOE*-containing phenotypes differently to the other authors. Where necessary, data were presented with or without the inclusion of these two studies to see if the findings were substantially changed.

The scales used to assess functional outcome of stroke were variable, including the BI which assesses activities of daily living (range 0–100), mRS assesses global disability (range 0–6), GOS assesses the degree of recovery (range 1–5) and NIHSS assesses stroke severity (range 0–42). NIHSS and GOS are usually used immediately whilst mRS and BI are used in the days to months after stroke [10, 11]. As a result, only a handful of studies have been conducted to equate these scales [12–14]. In the present study, we relied on the authors’ interpretation of ‘favourable’ or ‘poor’ outcome based on the particular scale used in their studies. The majority of investigators defined ‘favourable’ outcome as mRS of 0–1 or 0–2 whilst some [8] defined ‘favourable’ outcome as being one in which the patient was at home at follow-up (mRS = 0–4) and ‘poor’ if dead or in care (mRS = 5–6). Similarly, the BI was defined as follows: ‘favourable’ = 60–100 and ‘poor’ = 0–90, NIHSS score: ‘favourable’ = 0–10 and ‘poor’ = 2–42 and GOS: ‘favourable’ = 5–8 and ‘poor’ = 1–4.

Analysis was performed using data analysed by Review Manager v5.3 (Copenhagen: The Nordic Cochrane Centre, The Cochrane Collaboration, 2014). Pooled odds ratios (OR) were calculated with 95% confidence intervals (CI) by the random effects model. Inter-study heterogeneity was examined by *I*^2^ index and studies were iteratively tested to reduce heterogeneity. Funnel plots were used to assess publication bias. Statistical significance threshold was accepted as *p* < 0.05.

## Results

**Fig. 1:**
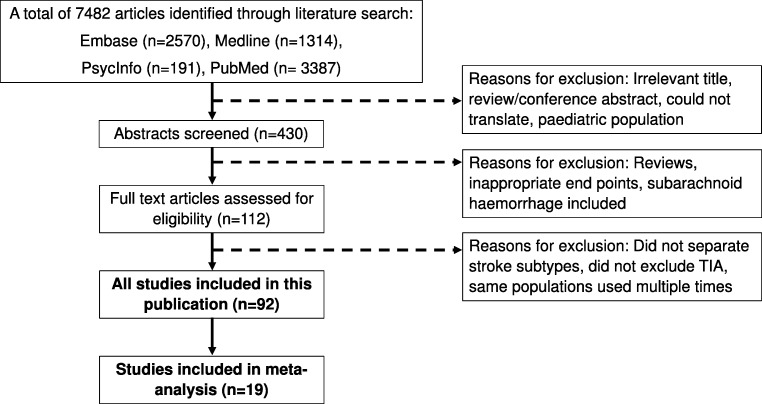
Flow chart of the screening process

Our initial search strategy found 7482 studies, and a total of 92 (comprising 135 polymorphisms in 72 genes) met inclusion criteria (Fig. 1) [8, 9, 15–104]. Characteristics of study populations are described in Supplementary Table 1. and summary of the studies examining the associations between genetic factors and stroke outcomes is shown in Supplementary Table 2.

### *APOE* variants on functional outcome several months following AIS

**Fig. 2:**
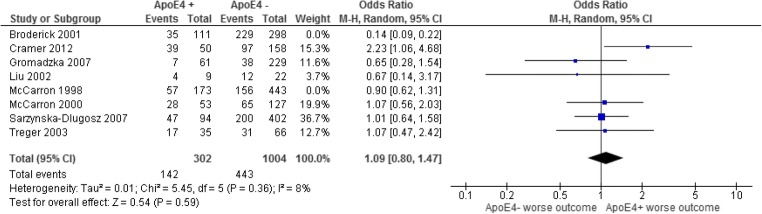
The odds ratio of having a poor functional outcome up to several months after an AIS with the presence of the ApoE4 allele compared with those without. The random effects model was used to determine the 95% confidence intervals and significance. The blue squares represent the individual studies results and weighting whilst the black diamond shows the overall result

Six studies [26, 43, 59, 67, 82, 90] comprising 2185 patients (490 with at least one *E4* allele and 1695 without) (Fig. 2) showed no association between outcome and the presence of at least one *E4* allele: OR = 1.02 (0.81–1.27, *p* = 0.90). There was no evidence of inter-study heterogeneity (*p* = 0.42, *I*^2^ = 1%) or publication bias. Including studies by McCarron et al. [8] and Broderick et al. [9] changed the OR to 0.78 (0.41–1.47, *p* = 0.44).

### *APOE* variants on functional outcome up to several months after an ICH

Three studies [26, 34, 51] comprising 118 patients (40 with at least one *E4* allele and 78 without) (Supplementary Fig. 1) revealed patients with ICH who possessed at least one *E4* allele had increased risk of poor outcome: OR 2.45 (1.03–5.81, *p* = 0.04). There was no evidence of inter-study heterogeneity (*p* = 0.70, *I*^2^ = 0%) or publication bias. Including the study by McCarron 1998 [8] changed the OR to 2.60 (1.25–5.41, *p* = 0.01).

### *BDNF-196* variants on functional outcome up to 7 years following AIS

**Fig. 3:**
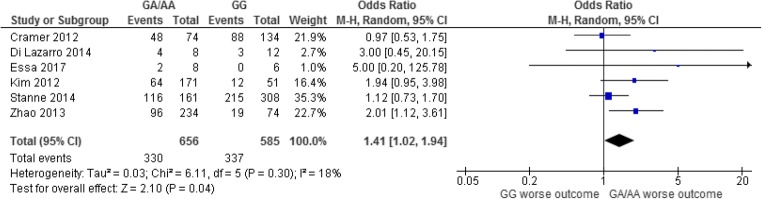
Outcome up to 12 months following an AIS with the GG variant at the BDNF-196 locus versus GA/AA

In six studies [26, 30, 33, 55, 89, 103] comprising 1241 cases (585 patients with a *GG* genotype and 656 without) (Fig. 3) showed patients with AIS who carried at codon 196 of the *BDNF*, the *GA* or *AA* genotypes were more likely to have poor outcome: OR 1.41 (1.02–1.94, *p* = 0.04). No heterogeneity (*p* = 0.30, *I*^2^ = 18%) or publication bias was observed. Excluding the study with longest follow-up of 7 years [89] increased the OR to 1.60 (1.08–2.37, *p* = 0.02).

### Cytochrome P450 2C19 (*CYP2C19*) loss of function variants on functional outcome at 6 months after an AIS

*CYP2C19* polymorphisms (poor metaboliser variants *CYP2C19* *2, *CYP2C19* *3 and *CYP2C19* *17) were studied in three studies comprising 918 patients (446 carried a loss of function allele and 472 without) (Supplementary Fig. 3). Patients with AIS who carried a loss of function *CYP2C19* allele were more likely to have poor outcome: OR = 2.36 (1.56–3.55, *p* < 0.0001). There was no significant heterogeneity (*p* = 0.96, *I*^2^ = 0%) or publication bias.

### Interleukin 6-174 (*IL-6*) variants on functional outcome at 6 months in AIS

Two studies totalling 237 patients (144 with the *GG* variant and 93 without) (Supplementary Fig. 4) found no association between patients who carried the *GC* or *CC* genotype at codon 176 of *IL6* gene and poor outcome: OR = 2.21 (0.55–8.86, *p* = 0.26).

## Discussion

In this meta-analysis, the largest to date, we have shown that genetic influences on functional outcomes in stroke recovery differed between patients who sustained ICH and those who sustained AIS. On the one hand, patients with ICH who possessed *APOE4* allele had a 2.6-fold increase in poor outcome; on the other hand, poor outcome was increased among patients with AIS who possessed poor metaboliser variants (*CYP2C19* *2, *CYP2C19* *3 and *CYP2C19* *17) by 2.4-fold and those who carried *BDNF GA* or *AA* genotypes by 1.6-fold, but not in patients with AIS who possessed *APOE4* or *IL6* alleles. Our findings suggest that genetic factors may, in part, account for the variability in stroke recovery and could be served as prognostic markers in the management of stroke.

It is unclear how *APOE4* influences poor outcomes in patients recovering from ICH but not in those recovering from AIS but may be linked to its relationship with coagulating ability. Weir et al. [105] have shown that among patients with ICH, compared with non-*APOE4* allele carriers, carriers of *APOE4* allele had higher partial thromboplastin time (PTT) ratios, i.e. greater propensity to bleed. The same study found that among patients with AIS, increasing APOE4 dose was associated with improved survival independent of stroke severity and PTT.

Similarly, underlying mechanisms on how *BDNF-196* variants influence outcomes in patient with AIS remain uncertain. Physical exercise in rehabilitation is thought to increase BDNF levels resulting in a greater capacity for neuronal survival and plasticity after stroke [103, 106]. However, a meta-analysis in non-stroke patients has shown a lack of association between *BDNF-196* genotype and serum BDNF levels [107]. Therefore, the association between *BDNF-196* variation and outcomes is more complex than first thought. The methylation status of the *BDNF* gene may be important [55, 108] and additionally any purported effects are likely to be due to the interplay of multiple polymorphisms at different loci [107].

We observed that loss of function of *CYP2C19* was also shown to associate with poor outcome in patient recovering from AIS. This relationship may be explained by a diminished ability of the enzyme to metabolise certain drugs used to treat AIS such as the antiplatelet agent clopidogrel [76, 100] resulting in reduced conversion of prodrug to the active form and efficacy. It has been shown that loss of function of *CYP2C19* allele is time dependent [52]; therefore, the adverse effects on stroke recovery may eventually wear off. However, all these studies were conducted in Chinese patients; therefore, these findings may not be applicable to other populations.

It appears that genes involved with drug metabolism such as *MDR1*, *COX2* and *CYP2C19* [52, 62, 71, 76, 83, 85, 100] appear to have major influences on stroke outcome which may be explained by the inflammatory cascade in the recovery process of AIS. Similarly, variants in IL-1, IL-4, IL-6, IL-10 and IL-12 have also been shown to associate with increased levels of inflammatory markers, tissue damage and worse outcomes [18, 22, 42, 66, 97]. In addition, PAI1, FXIII and tPA gene polymorphisms, known to involve in the clotting pathways, can also impact on the degree of thrombosis and the efficacy of fibrinolysis after stroke [36, 41, 62]. Many more mutations in other genes have been identified that have yet to converge with the existing literature.

### Limitations

As with all such analyses, a number of limitations need to be noted. Most papers focus solely on one or two genetic variations, yet it is likely that many such variants work in complex arrangements of gene-gene/gene-phenotype-environment interactions for eventual functional outcomes [100, 109]. For example, people with a family history of cardiovascular disease or risk factors such as hypercholesterolaemia and metabolic syndrome (increased central fat accumulation, hypertriglyceridaemia, low high-density lipoprotein cholesterol, hypertension and hyperglycaemia) may undergo behavioural changes such as intensive lifestyle modification.

The focus of our study was to examine the associations of genetic variants with functional outcomes in patients recovering from AIS or ICH. We recognise that the role of *APOE4* allele is more complex, involving small vessel disease, Alzheimer’s disease, risk of ICH recurrence and cerebral amyloid angiopathy-related syndromes [110]. However, this topic is beyond the aim of our present study.

Many of the genes described herein appear to correlate with the occurrence and severity of stroke. A more severe stroke usually leads to a worse outcome but this information was not available for our analysis whilst mortality was not consistently included in a number of studies; thus, heterogeneity in individual studies was unavoidable. Like all meta-analyses, methodologies between studies often vary; in the present study, we recognise variations in rehabilitation methods, outcome measures, duration of stroke recovery and definition of outcomes varied between papers may influence the degree of association between genetic mutations and stroke recovery but we do not feel the direction of association would be affected. To minimise the variability in outcome measures between studies, we have categorised them as consistently as possible.

The possibility of publication bias cannot be discounted, although funnel plots did not show asymmetrical distribution and we have performed exhaustive literature searches to ensure the maximum coverage. There may be heterogeneity or lack of agreement across scales such that some patients with identical BI scores can have very different mRS scores, highlighting the subjectivity of the mRS in which bias could potentially be introduced. Furthermore, scales tend to vary at different rates after stroke; therefore, combining scales may only be useful after a certain time, but the ‘optimal time’ remains undetermined. In contrast to most scales which consist of a wide range of score, mRS suffers from its narrow range of only seven points (0–6). Whilst Govan et al. [14] created an equivalency between the BI, mRS, NIHSS and Scandinavian stroke scale, the remaining studies focused mainly on mRS and BI. To provide a degree of flexibility when combining the scales in the present study, equivalent categories were created, e.g. a score of 0–3 on the mRS is equated to 10–20 on the BI.

In conclusion, we have demonstrated that recovery of AIS was unfavourably associated with variants of *BDNF* and *CYP2C19* genes whilst recovery of ICH was unfavourably associated with E4 allele of *APOE* gene. Our findings could be served as prognostic markers in the management of stroke.

## Electronic supplementary material

ESM 1 (DOCX 85 kb)

## Data Availability

No additional data are available.
