# Supplementary material for: Influences of genetic variants on stroke recovery: a meta-analysis of the 31,895 cases
Source: Neurol Sci. 2019 Jul 29;40(12):2437–45. doi: 10.1007/s10072-019-04024-w (PMC6848040; doi:10.1007/s10072-019-04024-w)

**Supplementary table 1.** Characteristics of the study populations.

| **Authors** | **Study year** | **Genes** | **Cases (n)** | **Mean age ±SD (range), years** | **Men (%)** | **Country** | **HTN (%)** | **DM (%)** | **Dyslipidaemia (%)** | **Smoking (%)** | **IHD (%)** | **AF (%)** |
| --- | --- | --- | --- | --- | --- | --- | --- | --- | --- | --- | --- | --- |
| **AIS** |  |  |  |  |  |  |  |  |  |  |  |  |
| Aberg | 2013 | *IGF1* | 600 | 56.7 | 64.2 | Sweden | 59.0 | 19.0 |  | 38.8 |  |  |
| Becker | 2014 | *IL1RN* | 113 | 55.5 | 66.4 | USA | 53.1 | 23.9 |  | 37.2 | 23.9 | 14.2 |
| Bouziana | 2018 | *Resistin* | 93 | 79.7 ±6.3 | 39.8 | Greece | 76.3 | 30.1 |  | 33.3 | 37.6 | 37.6 |
| Broderick | 2001 | *ApoE* | 27 |  |  | USA |  |  |  |  |  |  |
| Cai | 2017 | *PTGS1* | 617 | 58.72 | 73.9 | China | 70.2 | 26.7 |  | 39.4 |  |  |
| Chakraborty | 2013 | *IL6* | 100 | 54.0 ±10.9 (20-82) | 69.0 | India | 44.1 | 25.4 | 81.4 |  |  |  |
| Chang | 2017 | *BDNF* | 66 | 57.8 (29-80) | 54.5 | Korea |  |  |  |  |  |  |
| Cheng | 2018 | *ALDH2* | 1195 | 60.24 ±11.94 | 71.6 | China | 65.0 | 26.3 |  | 43.4 |  |  |
| Chon | 2016 | *BIRC5* | 121 | 65.74 ±12.12 | 55.3 | Korea | 45.1 | 14.5 | 30.4 | 40.5 |  |  |
| Díaz-Maroto Cicuéndez | 2017 | *UCP2* | 80 | 71.0 ± 12 (40-91) | 52.0 | Spain | 52.0 | 16.0 | 30.0 | 16.0 | 15.0 | 48.0 |
| Di Lazarro | 2014 | *BDNF* | 20 | 64.2 | 55.0 | Italy |  |  |  |  |  |  |
| Ellul | 2011 | *eNos* | 108 | 65.7 (34-93) | 68.0 | Greece | 72.0 | 26.0 | 72.0 | 42.0 | 21.0 |  |
| Essa | 2017 | *BDNF* | 14 | 64.9 | 64.3 | UK |  |  |  |  |  |  |
| Fernandez Cadenas | 2006 | *ApoE* | 77 | 71.4 | 44.6 | Spain | 44.2 | 21.4 | 33.3 | 18.6 | 22.2 | 40.0 |
| Fernandez Cadenas | 2010 | *PAI-1* | 165 | 70.8 | 48.3 | Spain | 49.3 | 21.4 | 31.2 |  |  |  |
| Fridriksson | 2018 | *BDNF* | 66 | 59.8 ±10.2 (30-77) | 69.7 | USA |  |  |  |  |  |  |
| Giannakopoulou | 2013 | *MCP1* | 145 | 67.0 ±12 | 65.5 | Greece | 72.0 | 28.0 | 70.0 | 36.0 | 26.0 | 21.0 |
| Gonzalez Conejero | 2006 | *FXIII* | 200 | 70.2 ±11.1 | 51.0 | Spain | 51.4 | 21.6 | 28.0 | 30.6 | 16.7 | 36.2 |
| Gromadzka | 2007 | *IL1RN* | 391 |  |  | Poland |  |  |  |  |  |  |
| Gromadzka | 2007 | *ApoE* | 666 | 71.0 ±12 | 50.0 | Poland | 68.0 | 17.6 |  | 45.2 | 32.7 | 23.7 |
| Gu | 2018 | *Chr10q25* | 739 |  | 71.6 | China | 92.7 | 32.5 | 42.1 | 42.8 | 7.8 | 3.1 |
| Guo | 2013 | *CRP* | 1680 | 61.2 | 61.9 | China | 47.6 | 26.1 | 13.9 | 30.2 |  | 14.9 |
| He | 2017 | *MTHFR* | 500 | 65.0 (51-77) | 66.6 | China | 44.2 | 31.8 |  | 64.2 |  |  |
| He | 2018 | *NOX4, MTHFR, NEIL3* | 308 | 65.81 ± 11.13 | 58.6 | China | 62.1 | 21.1 |  | 38.9 |  |  |
| Hoy | 2003 | *MPO* | 450 | 69, median (20-85) | 61.6 | France | 52.2 | 19.2 |  | 29.0 |  |  |
| Jia | 2013 | *CYP2C19* | 259 | 66.3 ± 11.8 | 64.5 | China |  | 37.3 | 26.3 |  |  |  |
| Jing | 2013 | *OPN* | 377 | 54.3 ± 7.1 | 0.6 | China | 49.6 | 43.8 |  | 35.0 |  |  |
| Keshavarz | 2016 | *BDNF* | 206 | 64.5 ± 11.7 | 51.9 | Iran | 73.3 | 40.8 | 39.3 |  | 37.9 |  |
| Kim | 2012 | *BDNF* | 286 | 64.5 ± 9.5 | 59.1 | Korea | 49.3 | 29.4 |  |  | 9.4 |  |
| Li | 2012 | *CX3CR1* | 308 | 63.6 ± 13 | 66.6 | China | 75.6 | 32.1 |  | 39.6 |  |  |
| Liu | 2002 | *ApoE* | 31 | 67.8 | 61.3 | Finland | 51.6 | 6.5 | 45.2 | 22.6 |  |  |
| Liepert | 2013 | *COMT* | 83 | 68.7 | 63.9 | Germany |  |  |  |  |  |  |
| Lovkvist | 2014 | *ApoD, SIGMAR1* | 3081 | 69, median (18-102) | 56.0 | Sweden | 97.8 | 96.7 |  | 98.3 |  |  |
| MacLeod | 2001 | *ApoE* | 266 | 65.7 ± 12.2 | 56.4 | UK | 44.7 | 9.0 |  |  |  | 10.5 |
| Maguire | 2011 | *GPIba, GPIIIa, tPa, PAI-1, COX2* | 615 | 74.9 ± 13.2 | 54.3 | Australia | 77.2 | 27.0 | 48.9 | 49.7 | 32.5 | 26.5 |
| Malueka | 2017 | *ACE* | 61 | 63.85 ± 13.1 | 59.0 | Indonesia | 73.8 | 37.7 | 19.7 | 41.0 | 9.8 |  |
| Marousi | 2011 | *TNFα, IL6, IL12B, IL4, IL10* | 145 | 68 median (58-76) | 65.5 | Greece | 72.0 | 28.0 | 70.0 | 36.0 | 26.0 | 21.0 |
| McCarron | 2000 | *ApoE* | 189 | 69.4 ± 11 (28-93) |  | UK | 42.0 | 12.0 | 16.0 | 35.0 | 31.0 | 16.0 |
| Mirowska Guzel | 2013 | *BDNF* | 46 | 62.1 ± 10 | 63.0 | Polish |  |  |  |  |  |  |
| Munshi | 2012 | *MDR1, LPL, eNos* | 525 | 49.3 ± 17.3 | 71.2 | India | 57.5 | 45.1 |  | 44.3 |  |  |
| Olsson | 2011 | *chr 9p21* | 111 | 60.0 ± 7 | 76.0 | Sweden | 65.0 | 34.0 |  | 53.0 |  |  |
| Qiu | 2015 | *CYP2C19* | 211 | 67.0 | 55.0 | China | 64.9 | 32.2 | 51.2 | 35.1 | 36.0 |  |
| Ramos-Araque | 2018 | *Tp53* | 60 | 72.64(±13.1) | 55.0 | Spain | 66.6 | 28.3 | 38.3 | 13.3 |  | 23.3 |
| Ritarwan | 2014 | *β fibrinogen* | 136 |  |  | Indonesia |  |  |  |  |  |  |
| Rogia | 2013 | *VKORC1* | 145 | 67.0 ± 12 | 65.5 | Greece | 72.0 | 28.0 | 70.0 | 36.0 | 26.0 | 21.0 |
| Sarzynska-Dlugosz | 2007 | *ApoE* | 496 |  | 48.0 | Poland | 70.0 | 17.0 |  | 45.0 | 33.0 | 25.0 |
| Sharma | 2012 | *MDR1* | 560 | 49.3 ± 17.3 | 70.2 | India | 57.2 | 45.1 |  | 44.3 |  |  |
| Sharma | 2013 | *ALOX5AP1* | 610 | 49.3 ± 17.3 | 68.0 | India | 57.2 | 45.1 |  | 44.3 |  |  |
| Sharma | 2013 | *COX2* | 450 | 49.3 ± 17.3 | 70.0 | India | 56.6 | 44.2 |  | 43.7 |  |  |
| Shi | 2018 | *MTHFR* | 400 |  | 69.0 | China | 77.8 | 60.2 |  | 75.2 |  |  |
| Song | 2017 | *PDE4D* | 1388 | 60.0 ± 3 | 75.1 | China | 61.5 | 28.8 | 16.1 | 60.5 | 10.2 | 3.7 |
| Stanne | 2014 | *BDNF* | 600 | 58, median (51-64) | 64.0 | Sweden | 59.0 | 19.0 | 69.0 | 39.0 |  |  |
| Treger | 2003 | *ApoE* | 101 |  |  | Israel |  |  |  |  |  |  |
| Wang | 2016 | *CYP2C19* | 321 | 62.0 (53-70) | 74.5 | China | 76.0 | 28.7 | 51.4 | 52.6 | 25.9 |  |
| Wang | 2019 | *HDAC9* | 1011 |  | 25.9 | China | 73.5 | 33.7 | 39.5 | 45.2 |  |  |
| Weinstein | 2014 | *TLR4* | 113 | 56.0 (42-67) | 66.4 | USA | 53.1 | 23.9 | 71.7 | 37.2 | 23.9 | 14.2 |
| Wu | 2016 | *Complement 5* | 494 | 69.8 ± 11.3 | 60.7 | China | 75.7 | 34.0 | 33.2 | 40.9 |  |  |
| Wzorek | 2018 | *α2AP* | 123 | 45.9 ± 11.4 | 35.0 | Poland | 28.0 | 7.0 | 25.0 | 24.0 |  |  |
| Yan | 2016 | *IL6* | 95 | 66, median (28-91) | 60.0 | Australia | 66.7 | 22.0 | 47.6 |  |  |  |
| Ye | 2018 | *CRP* | 690 | 61.1 ± 10.83 | 73.5 | China | 65.1 | 30.0 | 19.0 | 44.9 |  |  |
| Yi | 2016 | *CYP2C19, CYP3A4,CYP3A5* | 535 | 68.5 | 64.3 | China | 82.1 | 44.7 |  | 39.6 |  |  |
| Zhang | 2013 | *OLR1* | 304 | 61.2 ± 7.1 | 60.5 | China | 42.4 | 35.9 |  | 43.8 |  |  |
| Zhang | 2017 | *APOE* | 786 | 63.1 ± 11.6 (21-85) | 67.6 | China | 69.5 | 30.5 |  | 57.8 | 26.1 |  |
| Zhao | 2013 | *BDNF* | 494 | 69.8 ± 11.3 | 60.7 | China | 75.7 | 34.0 | 33.2 | 40.9 |  |  |
| Zhao | 2017 | *VEGF* | 494 | 69.8 ± 11.3 | 60.7 | China | 75.7 | 34.0 | 33.2 | 40.9 |  |  |
| **ICH** |  |  |  |  |  |  |  |  |  |  |  |  |
| Alberts | 1995 | *ApoE* | 44 |  | 50.0 | USA |  |  |  |  |  |  |
| Appelboom | 2013 | *PIK3CG, MRVI1, GP6* | 122 | 62.4 ± 17.6 | 58.2 | USA |  |  |  |  |  |  |
| Biffi | 2011 | *ApoE* | 2025 | 69.7 | 55.1 | Mixed | 67.3 |  |  |  |  |  |
| Dardiotis | 2008 | *ACT* | 147 | 63.9 ± 14.4 | 61.9 | Greece | 77.6 | 12.2 | 18.4 | 20.4 | 14.3 |  |
| El Husseini | 2018 | *IL6R, PARP1, IL6ST, TNF, MAPT, ApoE* | 54 | 66, median | 55.6 | USA | 88.9 | 24.1 |  |  | 9.6 | 14.8 |
| Fadel | 2012 | *ApoE* | 75 | 61.0 | 25.3 | Egypt | 76.0 | 44.0 | 22.7 | 37.3 | 29.3 |  |
| He | 2015 | *VEGF* | 244 | 62.0 ± 5.6 |  | China |  |  |  |  |  |  |
| James | 2009 | *ApoE* | 21 | 61.1 | 47.8 | USA |  |  |  |  |  |  |
| Marini | 2018 | *17p12* | 394 | 73.0 ± 10.0 | 53.0 | USA |  |  |  |  |  |  |
| Murthy | 2014 | *HP* | 94 | 59.5 | 53.2 | USA | 74.5 | 21.3 | 10.6 | 27.7 |  |  |
| Rodriguez | 2017 | *Tp53* | 78 | 70.4 ± 11.1 | 69.2 | Spain |  |  |  |  |  |  |
| Yang^‡^ | 2014 | *MMP9* | 169 | 61.8 | 74.0 | China | 85.8 | 7.1 | 3.0 | 27.8 |  |  |
| Xia^‡^ | 2018 | *COL4A1* |  |  |  |  |  |  |  |  |  |  |
| **Both AIS and ICH** |  |  |  |  |  |  |  |  |  |  |  |  |
| Cramer | 2012 | *BDNF, ApoE* | 255 | 68.9 | 56.5 | Mixed | 61.2 | 21.6 | 21.2 |  |  |  |
| de Boer | 2017 | *BDNF* | 40*, 13^†^ | 58.5 ± 10.6 | 68.0 | Netherlands |  |  |  |  |  |  |
| French | 2018 | *BDNF* | 63 (47*, 16^†^) | 61.1 ± 12.0 (25-86) | 66.7 | USA | 56.4 | 26.2 | 31.4 | 13.7 | 14.0 | 18.6 |
| Gomez Sanchez | 2011 | *Tp53* | 536 |  |  | Spain | 55.5 | 18.7 |  | 10.9 | 7.8 | 13.3 |
|  |  |  | 408* | 73.5 | 59.3 |  | 62.8 | 16.4 |  |  | 15.4 |  |
|  |  |  | 128^†^ | 72.6 | 57.8 | Portugal |  |  |  |  |  |  |
| Helm | 2016 | *BDNF* | 27 | 63.6 | 44.4 | USA |  |  |  |  |  |  |
| Lee | 2017 | *SERT* | 301 | 62.6 (52-73) | 62.1 | Korea | 73.8 | 38.9 | 48.5 | 48.5 | 16.6 |  |
| Manso | 2010 | *MMP2, MMP9* | 546 | 51.6 | 63.7 | Scotland | 42.0 |  |  | 58.0 | 26.0 |  |
| McCarron | 1998 | *ApoE* | 714 |  |  |  | 36.0 |  |  | 49.0 | 11.0 |  |
|  |  |  | 640* |  | 47.5 |  |  |  |  |  |  |  |
|  |  |  | 74† | 74, median (43-96) | 44.6 |  |  |  |  |  |  |  |
| Mirowska Guzel | 2012 | *BDNF* | 554 | 71.7 | 47.1 | Poland |  |  |  |  |  |  |
| Mirowska Guzel | 2014 | *BDNF* | 338 | 67.1 | 51.5 | Poland |  |  |  |  |  |  |
| Park | 2011 | *ITGA6* | 199 | 62.1 | 57.3 | Korea |  |  |  |  |  |  |
| Park | 2012 | *AGT* | 197 | 62.0 | 57.4 | Korea |  |  |  |  |  |  |
| Rodriguez | 2018 | *MDM2* | 408* | 72.8 ± 12.1 | 59.3 | Spain | 56.4 | 26.2 | 31.4 | 13.7 | 14.0 | 18.6 |
|  |  |  | 128* | 71.2 ± 12.3 | 57.8 |  | 55.5 | 18.7 |  | 10.9 | 7.8 | 13.3 |
| Shiner | 2016 | *BDNF, ApoE* | 54 | 61.4 ± 13.8 (18-83) | 75.9 | Australia |  |  |  |  |  |  |

HTN = hypertension, DM = Diabetes mellitus, IHD = Ischaemic heart disease, AF = atrial fibrillation.

*AIS patients, ^†^ICH patients. ^‡^same cohort.

**Supplementary table 2.** Table showing the results of the included studies.

| **Authors** | **Genes** | **Polymorphism** | **Genotype** | **Outcome time points** | **Outcome scales** | | | | |
| --- | --- | --- | --- | --- | --- | --- | --- | --- | --- |
|  |  |  |  |  | **mRs** | **m(BI)** | **NIHSS** | **GOS** | **Other** |
| **AIS** |  |  |  |  |  |  |  |  |  |
| Aberg | *IGF1* | *rs2162679, rs12821878, rs1019731, rs7956547, rs5742632, rs9989002, rs7136446, rs10860865, rs5742671, rs1520220, rs6214* | *With respect to major allele* | 3/24m* | 1.06, 1.14, 1.35, 1.05, 0.91, 1.15, 1.42, 1.05, 1.01, 1.03, 0.99 (OR) |  |  |  |  |
| Becker | *IL1RN* | *rs4251961* | *With respect to minor C allele* | 1/3/6/12m* | 7.67 (OR) |  |  |  |  |
| Bouziana | *Resistin* | *−420C>G* | *CC,CG,GG* | Discharge | 23/44, 27/39, 3/10 |  |  |  |  |
| Broderick | *ApoE* | *E4 +, E4 -* |  | 3m | 35/111, 229/298 | 18/111, 204/298 | 47/111, 244/298 | 28/111, 220/298 | |
| Cai | *PTGS1* | *rs1330344* | *TT, TC, CC* | 3m | 49/224, 61/289, 35/104 | |  |  |  |
|  |  | *rs3842788* | *GG, GA, AA* |  | 128/521, 17/93, 0/3 |  |  |  |  |
|  |  | *rs5788* | *CC, CA, AA* |  | 128/550, 17/63, 0/4 |  |  |  |  |
| Chakraborty | *IL6* | *IL6-174G/C* | *GG, GC, CC* | 7d, 3/6m | 17/56, 25/35, 3/7 | 10/53, 16/24, 3/6 |  |  |  |
| Cheng | *ALDH2* | *rs10744777* | *CC, CT/TT* | 3m | 293/1028, 23/89 |  |  |  |  |
|  |  | *rs886205* | *GG, GA, AA* |  | 246/887, 67/214, 2/12 | |  |  |  |
| Chon | *BIRC5* | *rs3764383* | *AA, AG, GG* | <1m |  | 41/55, 26/37, 4/4 | 30/62, 23/46, 4/5 | |  |
|  |  | *rs2071214* | *AA, AG, GG* | <1m |  | 32/45, 32/42, 7/9 | 28/54, 25/50, 4/9 | |  |
| Díaz-Maroto Cicuéndez | *UCP2* | *866G/A* | *AA, GA/GG* | 3m | 3/14, 43/66 |  |  |  |  |
| Di Lazarro | *BDNF* | *Val66Met (G196A) (rs6265)* | *VV, VM, MM* | <10 days |  |  | 3/12, 4/7, 0/1 |  |  |
| Ellul | *eNos* | *G894T* | *GT/TT vs GG* | 1m | 7.34 (OR) |  |  |  |  |
| Essa | *BDNF* | *Val66Met* | *VV, VM, MM* | 2 wks, 3m |  |  |  |  | 0/6, 2/7, 0/1 (DSRS) |
| Fernandez Cadenas | *BDNF* |  | *E2/2 + E2/3 + E2/4* | 2d, 3m | 1.5 |  |  |  |  |
|  |  |  | *E3/3 + E3/4 + E4/4* |  | 4 |  |  |  |  |
| Fernandez Cadenas | *PAI-1* | *RS1799768* | *4G4G, 4G5G/5G5G* | 1/2/12/24/48hr, 3m, Discharge |  |  | 15, 5 (median) | |  |
| Giannakopoulou | *MCP1* | *2518A>G* | *AA, GA/GG* | 1m | 46/77, 39/61 |  |  |  |  |
| Gonzalez Conejero | *FXIII* |  | *VV, VL/LL* | 1/2/12/24/48 hr, 3m, Discharge |  |  | 5,11 |  |  |
| Gromadzka | *IL1RN* |  | *IL1RN*1/1, 1/2, 2/2* | 7d, 1/3/12m | 50, 52, 42 (% with poor outcome) | 55, 62, 42% |  |  |  |
| Gromadzka | *ApoE* |  | *E2+, E3/3, E4+ (E2/4 absent)* | 1/3/12m | 4/39, 34/260, 7/61 | 7/39, 53/262, 9/62 |  |  |  |
| Gu | *Chr10q25* | *rs11196288A>G* | *AA, AG, GG* | 3m | 93/325, 93/332, 29/82 |  |  |  |  |
| Guo | *CRP* | *rs1130864* | *CC,CT,TT* | 3m | 499/1501, 76/177, 2/2 |  |  |  |  |
|  |  | *rs1800947* | *GG,GC,CC* |  | 513/1450, 58/216, 6/14 |  |  |  |  |
| He | *NOX4* | *rs11018628* | *TT, TC, CC* | Discharge |  |  | 101/210, 31/88,  3/10 |  |  |
|  | *MTHFR* | *rs1801133* | *TT, TC, CC* |  |  |  | 44/110, 59/132,  32/66 |  |  |
|  | *NEIL3* | *rs12645561* | *TT, TC, CC* |  |  |  | 14/32, 46/120,  75/156 |  |  |
| He | *MTHFR* | *rs868014* | *TT, TC, CC* | 3m |  |  | 0.7, 0.9,1.1 |  | 65.2,62.9,60.1 (FIM) |
| Hoy | *MPO* |  | *GG, AG/AA* | Before or at Discharge | 153/271, 128/194 |  |  |  |  |
| Jia | *CYP2C19* |  | *Poor metaboliser (LoF), intermediate/extensive* | 7d, 3/6m | 11/32, 31/174 |  |  |  |  |
| Jing | *OPN* | *OPN-443 C>T* | *TT, CT, CC* | 12m | 38/78, 67/135, 31/46 | 34/70, 72/138,  34/51 |  |  |  |
| Keshavarz | *BDNF* | *G196A* | *AA, GA/GG* | 6m |  | 86.35, 83.8 | 2.59, 2.2 |  |  |
| Kim | *IGF1* | *rs2162679* | *AA, AG, GG* | Unknown |  | 37/52, 29/38, 3/3 | 32/59, 20/46, 3/5 |  |  |
|  |  | *rs2195239* | *GG, GC, CC* |  |  | 25/33, 36/49, 10/13 | 16/39, 33/57,  8/16 |  |  |
|  |  | *rs978458* | *GG, GA, AA* |  |  | 24/31, 35/49, 12/15 | 15/37, 32/57,  10/18 |  |  |
|  |  | *rs1520220* | *CC, CG, GG* |  |  | 24/31, 35/49, 11/14 | 15/37, 31/57,  10/17 |  |  |
|  |  | *rs6214* | *GG, GA, AA* |  |  | 26/36, 37/45, 8/14 | 21/41, 26/53,  10/18 |  |  |
| Kim | *BDNF* | *Val66Met* | *VV, VM, MM* | 2wks, 12m | 21/60, 41/121, 23/50 |  |  |  |  |
| Li | *CX3CR1* | *V249I* | *VV, VI* | 1d, 2wks, 3m | 37/264, 1/17 |  |  |  |  |
|  |  | *T280M* | *TT, TM* |  | 36/258, 2/23 |  |  |  |  |
| Liu | *ApoE* |  | *E4+* | 1d, 3m | 4/9, (median=2) |  | (median=3) |  |  |
|  |  |  | *E4 -* |  | 12/22, (median=4) |  | (median=10) |  |  |
| Liepert | *COMT* | *rs4680 (Val158Met)* | *VV, VM, MM* | 4wks, 6m |  | ≈82, ≈82, ≈78 |  |  | ≈10, ≈10, ≈9 (Gross RMA) |
| Lovkvist | *ApoD* | *rs76929107, rs595, rs34697430, rs7659, rs823510* | *With respect to minor allele* | 3m | 0.92, 3.09, 0.92, 0.96, 0.97  (OR) |  |  |  |  |
|  | *SIGMAR1* | *rs11559048, rs1800866, rs12001648, rs7036351, rs3808873* |  |  | 2.29, 1.06, 0.75, 0.97, 1.07 |  |  |  |  |
| MacLeod | *ApoE* |  | *E2, E3, E4 (allele)* | Unknown | 0.08/0.07, 0.79/0.80, 0.13/0.13 (favourable/poor allele frequency) |  |  |  |  |
| Maguire | *COX2* | *rs5275* | *CC, CT, TT* | 3m* | 1.09, 1.54, 1 (OR) | 1.24, 1.53, 1 |  | 0.93, 1.43, 1 |  |
|  | *COX2* | *rs20417* | *CC, CG, GG* |  | 3.87, 1.35, 1 | 3.12, 0.93, 1 |  | 0, 1.81, 1 |  |
|  | *GPIIIa* | *rs5918* | *A1/1, A1/2, A2/2* |  | 1, 0.72, 0.67 | 1, 0.57, 0.52 |  | 1, 0.43, 0.83 |  |
|  | *GPIba* | *rs6065* | *TT, TM, MM* |  | 1, 1.04, 0 | 1, 0.68, 0 |  | 1, 0.91, 0 |  |
|  | *GPIba* | *rs2243093* | *TT, CT, CC* |  | 1, 1.16, 0.45 | 1, 0.89, 1.15 |  | 1, 0.84, 0.43 |  |
|  | *PAI-1* | *rs72578597* | *4G4G, 4G5G, 5G5G* |  | 1, 1, 1.21 | 1, 1, 0.89 |  | 1, 1.03, 1.16 |  |
|  | *tPA* | *rs63020761* | *CC, CT, TT* |  | 1, 1.14, 1.22 | 1, 1.09, 0.98 |  | 1, 1.07, 0.85 |  |
| Malueka | *ACE* | *I/D* | *II, ID/DD* | Discharge |  | 75, 60.65 |  |  |  |
| Marousi | *TNFα* | *TNFα-308G>A* | *AG, GG* | 6m |  | 13/28, 38/111 |  |  |  |
|  | *IL6* | *IL6-174G/C* | *GG, GC/CC* |  |  | 34/88, 21/51 |  |  |  |
|  | *IL12* | *IL12B-1188A>C* | *AA/CC, AC* |  |  | 37/85, 14/54 |  |  |  |
|  | *IL4* | *IL4-589C>T* | *CC, CT* |  |  | 43/114, 8/22 |  |  |  |
|  | *IL10* | *IL10-1082G>A* | *GG, AG/AA* |  |  | 12/26, 39/113 |  |  |  |
| McCarron | *ApoE* |  | *E2+, E3/3, E4+* | 1/3m | 30/53, 58/105, 8/23 |  |  |  |  |
| Munshi | *MDR1* | *C3435T* | *CC, CT, TT* | 12m | 43/103, 177/236, 144/186 |  |  | 43/103, 177/236,  144/186 |  |
|  | *LPL* | *HindIII* | *Hind -/-, -/+, +/+* |  | 23/48, 140/200, 195/278 |  |  | 23/48, 140/200,  195/278 |  |
|  | *eNos* |  | *4b4b, 4a4b, 4a4a* |  | 175/253, 149/216, 40/58 |  |  | 175/253, 149/216,  40/58 |  |
| Olsson | *chr9p21* | *rs7857345, rs1537378* | *With respect to minor allele* | 3m* | 0.25, 0.29, (OR) (LVD only) |  |  |  |  |
| Qiu | *CYP2C19* |  | *LoF carrier, normal* | 6m | 30/125, 8/73 |  |  |  |  |
| Ramos Araque | *Tp53* | *Arg72Pro* | *ArgPro/ProPro, ArgArg* | 3m | 2, 3 (median) |  |  |  |  |
| Ritarwan | *β fibrinogen* | *455 G/A* | *GG, GA, AA* | 1d, 2wks, 3m | 1/90, 0/37, 0/9 | 45/90, 37/61, 9/14 |  |  |  |
| Rogia | *VKORC1* | *1639G>A* | *GG/GA, AA* | 1m | 67/104, 18/34 |  |  |  |  |
| Sarzynska Dlugosz | *ApoE* |  | *E3/3, E3/4, E2/3, E2/4* | 12m | 170/304, 42/75, 30/53, 5/7 |  |  |  |  |
| Sharma | *MDR1* | *C3435T* | *CC, CT, TT* | 12m | 49/103, 107/256, 66/201 |  |  | 49/103, 107/256,  66/201 |  |
| Sharma | *ALOX5AP1* | *SG13S114T/A* | *TT, TA, AA* | 12m | 73/192, 148/285, 86/133 |  |  |  |  |
| Sharma | *COX2* | *rs20417* | *GG, GC, CC* | 3m | 80/201, 101/203, 36/46 |  |  |  |  |
| Shi | *MTHFR* | *rs142884651* | *GG, GA, AA* | 3m |  |  | 1.2, 0.8, 0,6 |  |  |
| Song | *PDE4D* | *rs918592* | *CC, CT, TT* | 3m | 192/302, 431/698, 269/380 |  |  |  |  |
|  |  | *rs966221* | *AA, AG, GG* |  | 545/845, 312/477, 37/59 |  |  |  |  |
| Stanne | *BDNF* | *rs6265* | *GG, GA, AA* | <7d, 3/24m, 7yrs | 115/208, 108/151, 8/10 |  |  |  |  |
|  |  | *rs11030107* | *AA, AG, GG* |  | 205/276, 116/178, 21/29 |  |  |  |  |
|  |  | *rs11030119* | *GG, GA, AA* |  | 182/242, 135/201, 25/40 |  |  |  |  |
|  |  | *rs2049046* | *TT, TA, AA* |  | 82/124, 188/260, 72/99 |  |  |  |  |
| Treger | *ApoE* |  | *E4+, E4-* | Discharge |  |  | 17/35, 31/66 |  |  |
| Wang | *CYP2C19* |  | *LoF carrier, normal* | 3/6/12m | 1.484 (RR) |  |  |  |  |
| Wang | *HDAC9* | *rs2074633* | *TT, TC, CC* | 3m | 109/387, 115/480, 50/144 |  |  |  |  |
|  |  | *rs28688791* | *TT, TC, CC* |  | 102/368, 119/490, 53/153 |  |  |  |  |
| Weinstein | *TLR4* | *Asp299Gly and/or*  *Thr399Ile* | | 3m | 14.16 (OR) |  |  |  |  |
| Wu | *Complement 5* | *rs17611* | *AA, AG, GG* | 1d, 3m | 76/194, 35/107, 4/7 |  |  |  |  |
| Wzorek | *α2AP* | *Arg407Lys* | *AA, AL, LL* | Unknown | 42/91, 5/29, 0/3 |  |  |  |  |
| Yan | *IL6* | *IL6-174G/C* | *CC, GG, CG* | 1d, 6m |  | ≈95, ≈90, ≈100 |  |  |  |
| Ye | *CRP* | *rs876537* | *CC, CT, TT* | 3m | 42/133, 97/335, 57/323 |  |  |  |  |
|  |  | *rs2794520* | *CC, CT, TT* |  | 40/136, 97/331, 59/324 |  |  |  |  |
|  |  | *rs3093059* | *CC, CT, TT* |  | 114/459, 73/207, 9/24 |  |  |  |  |
|  |  | *rs7553007* | *AA, AG, GG* |  | 59/323, 95/333, 42/134 |  |  |  |  |
|  |  | *rs11265260* | *AA, AG, GG* |  | 114/461, 72/204, 10/25 |  |  |  |  |
| Yi | *CYP3A5* | *rs776746* | *AA, AG/GG* | 6m | 7/70, 111/444 |  |  |  |  |
|  | *CYP2C19* | *rs4244285* | *GG, AG/AA* |  | 36/225, 82/289 |  |  |  |  |
| Zhang | *APOE* | *E4+* |  | Discharge | 300/670 |  | 88/670 |  |  |
|  |  | *E4-* |  |  | 57/116 |  | 11/116 |  |  |
| Zhang | *OLR1* | *rs11053646* | *GG, GC, CC* | 6m | 31/61, 70/126, 85/117 | 33/61, 65/126,  82/117 |  |  |  |
| Zhao | *BDNF* | *G196A* | *GG, GA, AA* | 1d, 3m | 19/74, 62/160, 34/74 |  |  |  |  |
| Zhao | *VEGF* | *rs3025039* | *CC, CT, TT* | 1d, 3m | 26/97, 60/145, 29/66 |  |  |  |  |
| **ICH** |  |  |  |  |  |  |  |  |  |
| Alberts | *ApoE* |  | *E2/2, 2/3, 2/4, 3/3, 3/4, 4/4* | Unknown |  | 90, 97, 100, 83,  60, 95 |  |  |  |
| Appelboom | *PIK3CG* | *rs342286* |  | 2wks or discharge | 0.495 (univariate association with poor outcome) |  |  |  |  |
|  | *MRVI1* | *rs7940646* |  |  | 0.281 |  |  |  |  |
|  | *GP6* | *rs1671152* |  |  | 0.097 |  |  |  |  |
| Biffi | *ApoE - Lobar* |  | *E2+, E4+* | 3m | 1.52, 1.08 (OR) |  |  |  |  |
|  | *ApoE - Deep* |  | *E2+, E4+* |  | 0.87, 1.61 |  |  |  |  |
| Dardiotis | *ACT* | *A/T* | *AA, TT/TA* | 6m | 63/110, 19/37 |  |  |  |  |
| El Husseini | *IL6R* | *rs2228145* | *With respect to major A allele* | 6m | 0.3 |  |  |  |  |
|  | *PARP1* | *rs3219119* | *With respect to major A allele* |  | 0.63 |  |  |  |  |
|  | *IL6ST* | *rs1900173, rs10940495* | *With respect to major A*  *allele* |  | 2, 0.16 |  |  |  |  |
|  | *TNFα* | *rs1800629, rs361525* | *With respect to major A allele, with respect to major G allele* |  | 0.99, 1.48 |  |  |  |  |
|  |  |  |  |  |  |  |  |  |  |
|  | *MAPT* | *rs2258689, rs10445337* | *With respect to major C*  *allele* |  | 2.67, 0.48 |  |  |  |  |
|  | *ApoE* | *rs429358, rs7412* | *With respect to major C*  *allele* |  | 1.71, 2.95 |  |  |  |  |
| Fadel | *ApoE* |  | *E4+, E4-* | 3m | 11/19/12/31 |  |  |  |  |
| He | *VEGF* | *rs3025020* | *TT, CT/CC* | 4wks |  | 34.8, 35.2 |  |  | 34.6, 33.6 (FM) |
|  |  | *rs3025039* | *TT, CT/CC* |  |  | 62.3, 70.5 |  |  | 58.5, 64.6 |
| James | *ApoE* |  | *E4+, E4-* | Discharge | 9/11, 4/10 |  |  | ≈2.75, ≈3.75 |  |
| Marini | *17p12* | *rs11655160* | *With respect to G allele* | 3m | 1.94 (OR) |  |  |  |  |
| Murthy | *HP* |  | *Hp1-1, 1-2, 2-2* | 3m | 8/12, 45/47, 32/36 |  |  |  |  |
| Rodriguez | *Tp53* | *Arg72Pro* | *ArgArg, ArgPro/ProPro* | 3/12m | 5, 0 (median) |  |  |  |  |
| Xia | *COL4A1* | *rs3742207* | *AA, AC, CC* | 6m | 44/108, 24/55, 3/6 |  |  |  |  |
|  |  | *rs11069830* | *AA, AC, CC* |  | 27/64, 35/83, 8/21 |  |  |  |  |
|  |  | *rs3783107* | *CC, CT, TT* |  | 18/47, 33/84, 20/37 |  |  |  |  |
|  |  | *rs532625* | *AA, AT, TT* |  | 18/30, 35/91, 16/46 |  |  |  |  |
|  |  | *rs544012* | *AA, AC, CC* |  | 16/29, 35/89, 17/34 |  |  |  |  |
|  |  | *rs679505* | *AA, AG, GG* |  | 18/47, 34/73, 19/48 |  |  |  |  |
| Yang | *MMP9* | *rs3918241* | *AA, AT, TT* | 3m | 2/4, 14/37, 55/118 |  |  |  |  |
|  |  | *rs1805088* | *CC, CT, TT* |  | 72/165, 2/4, 0/0 |  |  |  |  |
|  |  | *rs17576* | *AA, AG, GG* |  | 2/10, 37/68, 35/91 |  |  |  |  |
|  |  | *rs3918254* | *CC, CT, TT* |  | 51/106, 23/59, 0/4 |  |  |  |  |
|  |  | *rs3787268* | *AA, AG, GG* |  | 11/23, 40/80, 21/59 |  |  |  |  |
|  |  | *rs17577* | *AA, AG, GG* |  | 2/5, 15/38, 57/126 |  |  |  |  |
|  |  |  |  |  |  |  |  |  |  |
| **Both AIS and ICH** |  |  |  |  |  |  |  |  |  |
| de Boer | *BDNF* | *G196A* | *VV* | Unknown |  |  |  |  | 35.2 (ANELT) 28.8 (BNT) |
|  |  |  | *VM/MM* |  |  |  |  |  | 37.7, 28.9 |
| Chang | *BDNF* | *G196A* | *VV, VM/MM* | 3m |  |  |  |  | 9/14, 45/52 (FM-Upper Limb) |
| Cramer | *BDNF** | *rs6265* | *VV, VM, MM* | 1/3m | 88/134, 45/65, 3/9 |  |  |  |  |
|  | *BDNF*^†^ |  |  |  | 19/24, 15/20, 1/3 |  |  |  |  |
|  | *ApoE** |  | *E4-, 1x E4, E4/4* |  | 97/158, 37/47, 2/3 |  |  |  |  |
|  | *Apoe*^†^ |  |  |  | 27/37, 8/10, 0/0 |  |  |  |  |
| French | *BDNF* | *G196A* | *Met + vs Met -* | Unknown |  |  |  |  | ΔR2 = 0.012 (Regression)  (10 metre walk test) |
| Fridriksson | *BDNF* | *G196A* | *VV, VM/MM* | Unknown |  |  | 4.8, 5.2 |  |  |
| Gomez Sanchez | *Tp53* | *Arg72Pro** | *ArgArg, ArgPro, ProPro* | 3m | 131/235, 30/147, 0/26 | |  |  |  |
|  |  | *Arg72Pro*^†^ |  |  | 54/67, 5/54, 0/7 |  |  |  |  |
| Helm | *BDNF* | *Val66Met* | *VV, VM/MM* | Unknown |  |  |  |  | 22.5, 23.3 (FM-Lower Limb) |
| Lee | *SERT* | *STin2 VNTR* | *10-12, 12-12* | 6m | 26/56, 146/245 |  |  |  |  |
|  |  | *5-HTTLPR* | *II, IS, SS* |  | 7/15, 61/106, 104/180 |  |  |  |  |
| Manso* | *MMP2* | *rs243866* | *GG, GA, AA* | 3m | 83/208, 55/114, 8/9*  34/51, 21/28, 1/2^†^ |  |  |  |  |
|  |  | *rs243865* | *CC, CT, TT* |  | 83/207, 55/113, 8/9*  34/51, 21/28, 1/2^†^ |  |  |  |  |
|  |  | *rs857403* | *AA, TA, TT* |  | 103/208, 37/107, 6/15*  35/50, 19/24, 2/3^†^ |  |  |  |  |
|  |  | *rs1477017* | *AA, GA, GG* |  | 55/141, 72/154, 19/36*  26/40, 26/35, 4/6^†^ |  |  |  |  |
|  |  | *rs17301608* | *CC, CT, TT* |  | 50/130, 73/158, 22/41*  25/39, 27/36, 4/6^†^ |  |  |  |  |
|  |  | *rs1053605* | *CC, CT, TT* |  | 127/293, 16/35, 3/3*  43/65, 12/15, 1/1^†^ |  |  |  |  |
|  |  | *rs2241145* | *GG, GC, CC* |  | 39/107, 72/160, 34/62*  17/28, 29/41, 10/12^†^ |  |  |  |  |
|  |  | *rs243849* | *CC, CT, TT* |  | 39/107, 72/160, 34/62*  17/28, 29/41, 10/13^†^ |  |  |  |  |
|  |  | *rs183112* | *GG, AG, AA* |  | 39/107, 72/160, 34/62*  17/28, 29/41, 10/14^†^ |  |  |  |  |
|  |  | *rs1992116* | *GG, AG, AA* |  | 39/107, 72/160, 34/62*  17/28, 29/41, 10/15^†^ |  |  |  |  |
|  | *MMP9* | *rs8113877, rs3918253, rs2236416, rs17577* |  |  | 39/107, 72/160, 34/62*  17/28, 29/41,  10/16† |  |  |  |  |
| McCarron | *ApoE** |  | *E4+, E4-* | 3m | 57/173, 156/443 |  |  |  |  |
|  | *ApoE*^†^ |  | *E4+, E4-* |  | 13/16, 30/51 |  |  |  |  |
| Mirowska Guzel | *BDNF* | *G196A** | *GA/AA, GG* | 7d, 1m, Discharge | 2, 2 (median) |  |  |  |  |
|  |  | *G196A*^†^ | *GA/AA, GG* |  | 4, 2.5 |  |  |  |  |
|  |  | *270C/T** | *CT, CC* |  | 2.5, 2 (median) |  |  |  |  |
| Mirowska Guzel | *BDNF** | *G196A* | *GA/AA, GG* | Discharge |  |  | 4.41, 4.93 |  |  |
| Park | *ITGA6* | *rs11895564* | *GG, GA, AA* | Unknown |  | 62/78, 9/18, 0/0* | 40/58, 9/13, 1/3^†^ |  |  |
|  |  | *rs2293649* | *GG, GA, AA* |  |  | 19/29, 36/47, 16/20* | 11/22, 24/35,  15/17^†^ |  |  |
| Park | *AGT*^†^ | *rs4762* | *GG, GA, AA* | Unknown |  |  | 31/52, 16/18, 2/3 |  |  |
|  |  | *rs699* | *GG, GA, AA* |  |  |  | 35/51, 12/19, 2/3 |  |  |
| Rodriguez | *MDM2* | *309T>G** | *TT, TG, GG* | 12m | 3,2,1 (median) |  |  |  |  |
|  |  | *309T>G*^†^ | *TT, TG, GG* |  | 4,1,2 |  |  |  |  |
| Shiner | *BDNF* | *rs6265* | *VV, VM/MM* | Unknown |  |  |  |  | 42.0, 46.1 (FM-Upper Limb) |
|  | *ApoE* |  | *E4+, E4-* |  |  |  |  |  | 50.0, 42.8 |

mRS = modified Rankin score, m(BI) = modified (Barthel Index), NIHSS = National Institutes of Health Stroke Scale, GOS = Glasow Outcome Scale, ‘Other’ category includes dysphagia severity rating scale (DSRS), FIM = functional Independence Measure, FM = Fugl-Meyer, BNT = Boston Naming Test, ANELT = Amsterdam Nijmegen Everyday Language Test, RMA = Rivermead motor assessment. Results given as number of patients with poor outcome/total unless otherwise stated. ‘Outcome timepoints’ = different times the authors recorded results, but only the latest time point was detailed here.*AIS patients; ^†^ICH patients.

**Supplementary figure 1.** The odds ratio of having a poor functional outcome up to several months after an ICH with an ApoE4 allele versus without one. The random effects model was used to determine the 95% confidence intervals and significance. The blue squares represent the individual studies results and weighting whilst the black diamond shows the overall result.


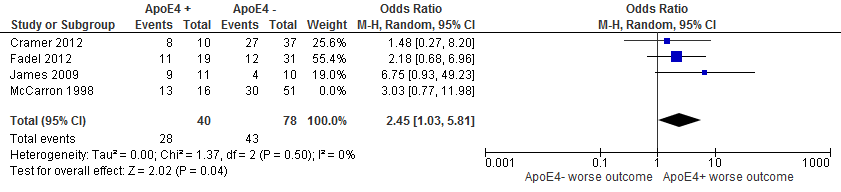


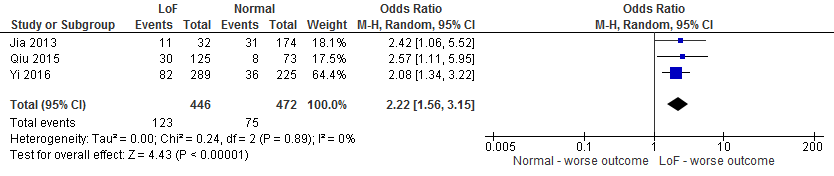
**Supplemental figure 2.** The odds ratio of having a poor functional outcome at 6 months after an AIS with a loss of function (loss of function) allele of CYP2C19 versus without (normal). The random effects model was used to determine the 95% confidence intervals and significance. The blue squares represent the individual studies results and weighting whilst the black diamond shows the overall result.

**Supplementary figure 3.** The odds ratio of having a poor functional outcome at 6 months with the GG variant of IL6-174 versus GC/CC. The random effects model was used to determine the 95% confidence intervals and significance. The blue squares represent the individual studies results and weighting whilst the black diamond shows the overall result.


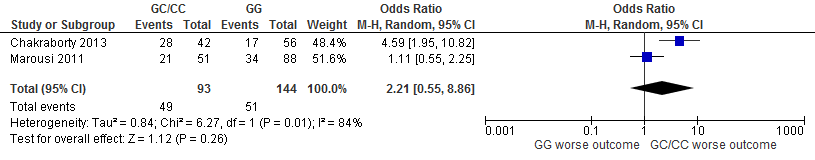

Supplement: Supplementary file 1 — (DOCX 85 kb) [file 10072_2019_4024_MOESM1_ESM.docx]
